# Identification of gene expression profiles in myocardial infarction: a systematic review and meta-analysis

**DOI:** 10.1186/s12920-018-0427-x

**Published:** 2018-11-27

**Authors:** Panagiota Kontou, Athanasia Pavlopoulou, Georgia Braliou, Spyridoula Bogiatzi, Niki Dimou, Sripal Bangalore, Pantelis Bagos

**Affiliations:** 1 Department of Computer Science and Biomedical Informatics, University of Thessaly, 35131 Lamia, Greece; 2 Izmir Biomedicine and Genome Institute, Dokuz Eylül University Health Campus, 35340 Izmir, Turkey; 3 Department of Hygiene and Epidemiology, University of Ioannina School of Medicine, Stavros Niarchos Av, 45110 Ioannina, Greece; 4 School of Medicine, New York University, New York, NY 10016 USA; 5 Lamia, University of Thessaly, Papasiopoulou 2-4, 35131 Lamia, Greece

**Keywords:** Myocardial infarction, Gene-expression, Meta-analysis, Differentially expressed genes, Biomarkers, Risk prediction

## Abstract

**Background:**

Myocardial infarction (MI) is a multifactorial disease with complex pathogenesis, mainly the result of the interplay of genetic and environmental risk factors. The regulation of thrombosis, inflammation and cholesterol and lipid metabolism are the main factors that have been proposed thus far to be involved in the pathogenesis of MI. Traditional risk-estimation tools depend largely on conventional risk factors but there is a need for identification of novel biochemical and genetic markers. The aim of the study is to identify differentially expressed genes that are consistently associated with the incidence myocardial infarction (MI), which could be potentially incorporated into the traditional cardiovascular diseases risk factors models.

**Methods:**

The biomedical literature and gene expression databases, PubMed and GEO, respectively, were searched following the PRISMA guidelines. The key inclusion criteria were gene expression data derived from case-control studies on MI patients from blood samples. Gene expression datasets regarding the effect of medicinal drugs on MI were excluded. The t-test was applied to gene expression data from case-control studies in MI patients.

**Results:**

A total of 162 articles and 174 gene expression datasets were retrieved. Of those a total of 4 gene expression datasets met the inclusion criteria, which contained data on 31,180 loci in 93 MI patients and 89 healthy individuals. Collectively, 626 differentially expressed genes were detected in MI patients as compared to non-affected individuals at an FDR q-value = 0.01. Of those, 88 genes/gene products were interconnected in an interaction network. Totally, 15 genes were identified as hubs of the network.

**Conclusions:**

Functional enrichment analyses revealed that the DEGs and that they are mainly involved in inflammatory/wound healing, RNA processing/transport mechanisms and a yet not fully characterized pathway implicated in RNA transport and nuclear pore proteins. The overlap between the DEGs identified in this study and the genes identified through genetic-association studies is minimal. These data could be useful in future studies on the molecular mechanisms of MI as well as diagnostic and prognostic markers.

**Electronic supplementary material:**

The online version of this article (10.1186/s12920-018-0427-x) contains supplementary material, which is available to authorized users.

## Background

Atherosclerotic heart disease is manifested by atherosclerosis and has a broad underlying pathophysiological spectrum. It comprises, among others, ischemic heart disease (IHD), coronary artery disease (CAD), stroke, and myocardial infarction (MI), commonly known as heart attack. Atherosclerotic heart diseases represent the leading cause of morbidity and mortality globally, accounting for 17.3 million deaths per year [1, 2], resulting to approximately one-third of all deaths worldwide [3, 4]. CAD is a group of diseases including stable angina, acute coronary syndrome, and sudden cardiac death; the most important complication of CAD is MI [4]. CAD and MI are complex and multifactorial diseases that are attributed to the interaction of both genetic and environmental factors [5, 6]. Traditional risk factors include smoking, physical inactivity and obesity, as well as disorders such as diabetes, hypertension and dyslipidemia [7]. Cholesterol and lipid metabolism has attracted particular interest from the researchers in the field of cardiovascular diseases. The molecular mechanisms that have been proposed thus far to underlie the pathogenesis of MI, apart from those related to cholesterol and lipid metabolism, include mechanisms related to the regulation of thrombosis and inflammation [8–10]. More recently, emerging roles have been also attributed to oxidative stress and DNA damage [7].

Genome-wide association studies have revealed a great number of inter-individual genetic variations associated with MI, such as single nucleotide polymorphisms (SNPs) (http://www.cardiogramplusc4d.org/). This enabled the development of genetic risk scores to be used in parallel with traditional cardiovascular risk scores such as Framingham score [11]. Large-scale gene expression profiling with microarrays technology has enabled the prediction of other disease states such as precancerous condition [12] or increased oxidation and inflammation state in sickle cell disease patients [13]. Nowadays, there is an increasing interest in identifying gene expression profiles based principally on microarrays (transcriptomics) for the diagnosis of MI, as well as for risk prediction of MI and cardiovascular death [14, 15].

The purpose of this study was to collect the available expression data on differentially expressed genes (DEGs) that are consistently associated with the incidence of MI and identify key components of the molecular pathways involved in the pathogenesis of the disease. Such analyses could also be useful in identifying key genes whose differential expression can be used for disease diagnosis and prognosis. Towards this end, gene expression data from case-control studies in MI were retrieved from multiple, independent microarray studies and a carefully designed meta-analysis was performed following the guidelines.

## Methods

In order to identify gene expression data regarding myocardial infarction, we performed a comprehensive literature search in PubMed [16] using the keywords “microarray” AND (“myocardial ischaemia” OR “myocardial infarction”). The datasets were retrieved from the public microarray data repository GEO [17], using the search term “myocardial infarction”. Datasets that include gene expression data on tissues other than blood, as well as datasets regarding the effect of drugs in the above mentioned diseases, were excluded from our analysis. Studies that met the inclusion criteria but did not make their data available could not be included in the meta-analysis, but nevertheless they are included in the systematic review. The overall procedure of data extraction is shown in the PRISMA Flow Diagram (Additional file 1: Figure S1).

For each microarray study, we recorded the gene expression data matrix that represents the gene expression summary for every probe and every sample and used it as input to the meta-analysis. In microarrays, especially when combining data from different platforms which use different probes, several problems may occur. Many probes can map to the same Gene ID for various reasons, and, conversely, a probe may also map to more than one Gene ID if the probe sequence is not specific enough. A simple approach would be to use only the probes with one-to-one mapping for further analysis; however, this approach results to loss of information. To circumvent this, and in order to perform an analysis based on genes and not probes, we followed the guidelines of Ramasamy and coworkers and we converted the probe identifiers to gene identifiers before conducting meta-analysis. To this end, GPL files that contained infromation about the gene symbols that correspond to probe id’s were used in order to combine studies from different platforms and resolve the “many-to-many” relationships between probes and genes, by averaging the expression profiles for genes with more than one probe [18].

The *t*-test was employed to identify the differentially expressed genes (DEGs) between the case and control groups. A drawback of the *t*-test in microarray data analysis is that in case most of the experiments in a study contain only few samples in each group the assumption of normality is not tenable. To resolve this, Bootstrap [19, 20], a statistical method for estimating the sampling distribution of an estimator by resampling with replacement from the original sample was used. Bootstrap provides an ideal alternative method when no formula for the sampling distribution is available or when the available formulas make inappropriate assumptions (e.g. small sample size, non-normal distribution). The Bootstrap method has been applied in previous microarray experiments, and empirical evidence suggests that it produces accurate estimates, at least for moderate sample sizes [21]. For very small sample sizes (i.e. < 10), various modifications to the standard Bootstrap method have been proposed [22, 23]. Bootstrap analysis was conducted with 1000 replicates, a relatively high number, in order to generate acurate estimates of the standard errors.

The generated Bootstrap standard errors were subsequently used in a standard procedure for random effects meta-analysis by employing the standardized mean difference [24, 25]. In order to account for the multiple comparisons, various correction methods were considered in this study. These methods are grouped into two categories, the ones that control the family-wise error rate (FWER) and the ones that control the False Discovery Rate (FDR). The most common approach to control FWER is the Bonferroni correction [26] which is easily applied and intuitive, but it is very conservative. Other popular methods used for multiple testing correction are the methods proposed by Sidak [27], Holland et al. [28] and Holm [29]. Benjamini and Hochberg [30] proposed a method which controls FDR instead of FWER. FDR-controlling procedures are designed to control the expected proportion of rejected null hypotheses that were incorrect rejections. FDR-controlling procedures have greater power (i.e. they detect more differences as statistically significant), at the cost of increased rates of Type I errors. For both FWER and FDR analyses, genes with the FDR-corrected *p*-value (*q*-value) less or equal to 0.01 were considered as statistically significant. Finally, the integration-driven discovery rate (IDR) proposed previously [25, 31] was used in order to calculate the DE genes identified purely by the meta-analysis. The IDR is defined as the proportion of genes that are identified in the meta-analysis and were not identified in any of the individual studies, using the same statistical criteria. For all statistical analyses, the Stata v13 statistical software package [32] was used.

The identified differentially expressed genes were submitted to STRING v10 [33] for in silico gene/protein interaction analysis. STRING (Search Tool for the Retrieval of INteracting Genes/proteins) [33] is a comprehensive database of known and predicted, direct and indirect interactions among genes/proteins, derived from a variety of sources such as high-throughput biochemical, genetic or biophysical experiments, co-expression analyses, and others. Furthermore, statistically significant over-represented KEGG Pathway [34] terms were identified by employing WebGestalt (WEB-based GEne SeT AnaLysis Toolkit) [35]. Hypergeometric distribution analysis [36] was used and the *p-*values were adjusted with the FDR correction [30]; the threshold for *q*-values was set at 10^− 3^. A similar analysis was performed for genes which are known to have polymorphisms associated with CAD/MI (genetic association data). These genes were obtained by a previous comprehensive analysis [37, 38], which combined data from three diverse databases of 7158 gene-disease association data: the NCBI’s OMIM (Online Mendelian Inheritance in Man) [39], the NIH’s GAD [40] and the NHRI GWAS Catalog [41].

## Results

**Fig. 1:**
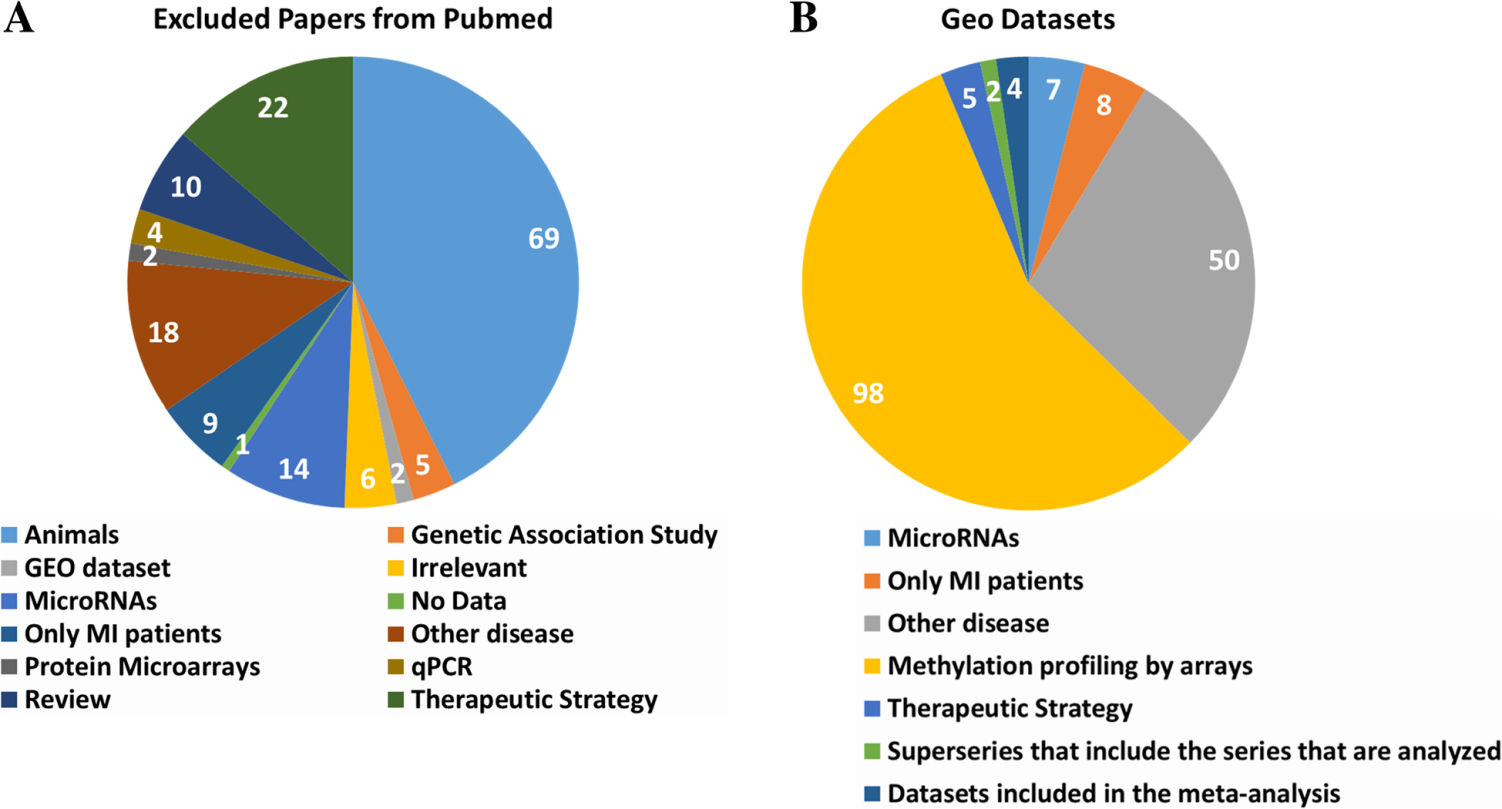
**a**) Articles screened from Pubmed database **b**) Datasets screened from Geo Database

A total of 162 articles and 174 datasets were retrieved from PubMed and GEO and reviewed for eligibility. The 160 articles from PubMed were irrelevant research articles or reviews and were subsequently excluded from the meta-analysis (Fig. 1A). Additional file 2 provides a detailed list of PMIDs for the articles identified and the reasons for their exclusion. Only one published paper could potentially meet the inclusion criteria but the authors did not make their data available in GEO (or any other database), so we could not include it in the meta-analysis. Finally, 2 articles identified by the literature search in Pubmed contain information on GEO three datasets that had already been identified and included in our analysis. Among the GEO datasets, four met the eligibility criteria and were included in the meta-analysis (Fig. 1B), (Additional file 1: Figure S1). These datasets contained data on 31,180 loci, in 93 patients with MI and 89 healthy individuals (Table 1). The paper published by Głogowska-Ligus and Dąbek (2012) [42] did not make the data available (and hence it could not be included in the meta-analysis), but the authors identified 26 DEGs (Additional file 1: Table S1). Among these genes only three (TKT, HCK, SERPINA1) were found among the results of the meta-analysis (see below).

**Table 1:** Study Characteristics included in meta-analysis

References	GEO Dataset	Platform	MI patients	Healthy controls	Number of probes	Number of Genes
[60]	GSE48060	Affymetrix Human Genome U133 Plus 2.0 Array	30	22	42,450	21,037
[61]	GSE60993	Illumina HumanWG-6 v3.0 expression beadchip	7	7	35,966	25,162
[61]	GSE61144	Sentrix Human-6 v2 Expression BeadChip	7	10	30,535	24,778
–	GSE66360	Affymetrix Human Genome U133 Plus 2.0 Array	49	50	42,450	21,037

**Fig. 2:**
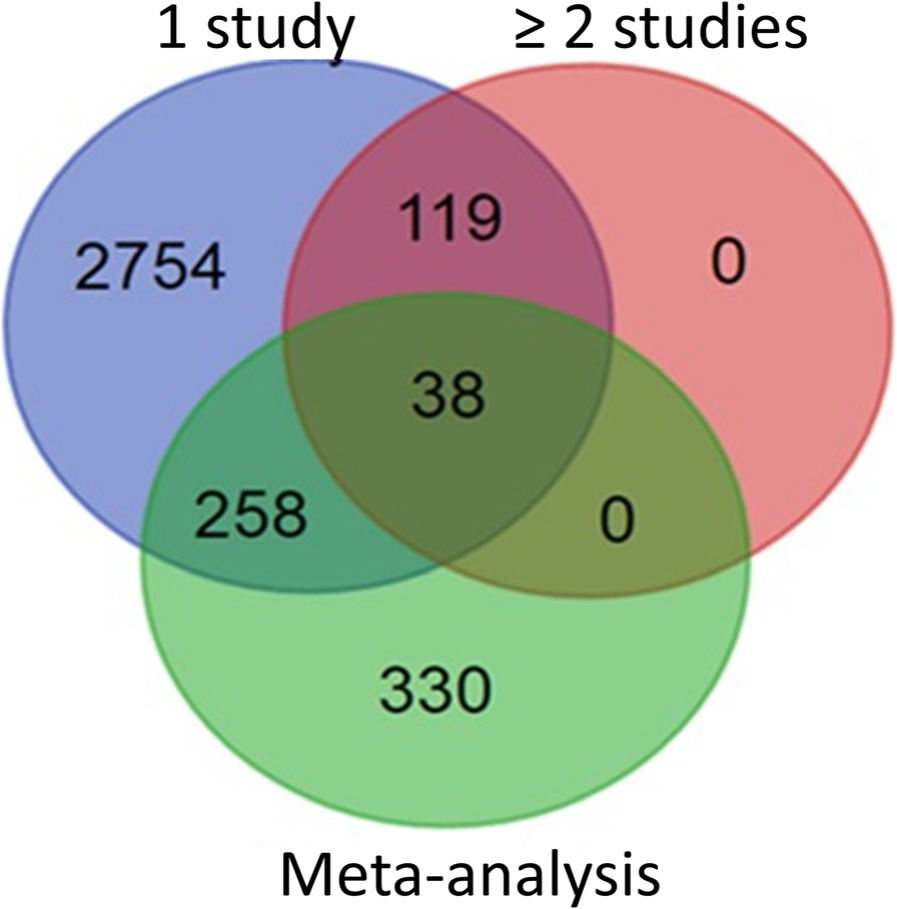
Venn diagram comparing DEG sets identified by the individual studies and by meta-analysis. The results obtained by the meta-analysis (626 DEGs) are compared with DEGs identified by at least one study and DEGs identified by at least two studies

In our meta-analysis, we identified a total of 626 differentially expressed genes in MI patients as compared to healthy individuals at an FDR-adjusted *p*-value threshold of 0.01 [30]. Several methods of multiple testing correction (Sidak [27], Bonferoni [26], Holm [29], Holland [28]) were applied in order to reduce the number of false positives. All FWER methods identified fewer genes as statistically significant (Additional file 1: Table S2). A gene found to be differentially expressed by meta-analysis can be likely not found to be DE in any of the individual studies (Fig. 2). In our study, the integration-driven discovery rate (IDR) was computed in order to determine the proportion of DEGs detected by meta-analysis as compared to the individual studies [25, 31]. The IDR was estimated to be 0.527, indicating that the percentage of DEGs identified through meta-analysis is 52.7%. The 626 DEGs with their FDRs are shown in Additional file 1: Table S3. These 626 differentially expressed genes were assessed for KEGG Pathway terms enrichment, but no enrichment could be found at *p*-value< 0.05. The top-60 genes at an FDR < 10^− 8^ are presented in Additional file 1: Table S4. The biological processes of the 626 DEGs, according to STRING, appear in Table 2A. Many genes participate in the broad categories of cellular and metabolic processes, and of particular note, 30 genes are involved in inflammatory responses while 12 in cytokine production. Moreover, a great number (i.e. 343) of the gene products are membrane-associated (in plasma membrane or organelles) (Τable 2B).

**Table 2:** Enrichment Analysis of the 626 DEGs according to STRING

A: Functional enrichment of the 626 DEGs for Biological Processes according to STRING			
	Biological Process (GO)		
pathway ID	pathway description	count in gene set	false discovery rate
GO:0009987	cellular process	363	5.80E-07
GO:0071704	organic substance metabolic process	265	0.000675
GO:0044237	cellular metabolic process	256	0.000684
GO:0008152	metabolic process	283	0.000785
GO:0006954	inflammatory response	30	0.00134
GO:0044238	primary metabolic process	253	0.00504
GO:0044699	single-organism process	296	0.0303
GO:0051186	cofactor metabolic process	21	0.0303
GO:0045321	leukocyte activation	25	0.0329
GO:1901564	organonitrogen compound metabolic process	66	0.0329
GO:0002274	myeloid leukocyte activation	11	0.0381
GO:0006807	nitrogen compound metabolic process	174	0.0381
GO:0001816	cytokine production	12	0.043
GO:0044763	single-organism cellular process	282	0.043
B: Cellular Component enrichment of the 626 DEGs for Cellular Component according to STRING			
Cellular Component (GO)			
pathway ID	pathway description	count in gene set	false discovery rate
G0:0044424	intracellular part	370	1.47E-05
GO:0005622	intracellular	376	1.59E-05
G0:0043227	membrane-bounded organelle	343	1.59E-05
G0:0043226	organelle	354	4.39E-05
G0:0043231	intracellular membrane-bounded organelle	309	0.000327
GO:0043229	intracellular organelle	326	0.000596
GO:0005623	cell	393	0.00139
G0:0044464	cell part	391	0.00195
G0:0005737	cytoplasm	294	0.00216
GO:0005575	cellular_component	423	0.00292
GO:0035859	Seh1-associated complex	4	0.0049
G0:0044194	cytolytic granule	3	0.0103
G0:0044444	cytoplasmic part	221	0.0137
G0:0061700	GATOR2 complex	3	0.0216
G0:0042581	specific granule	4	0.033
C: KEGG Pathway enrichment of the 88 DEGs that were strongly interconnected and formed a network according to STRING.			
#pathway ID	pathway description	observed gene count	false discovery rate
3050	Proteasome	6	0.000298
3013	RNA transport	8	0.00253
4144	Endocytosis	9	0.00253
564	Glycerophospholipid metabolism	6	0.00596
532	Glycosaminoglycan biosynthesis - chondroitin sulfate / dermatan sulfate	3	0.0256
4666	Fc gamma R-mediated phagocytosis	5	0.0256
5323	Rheumatoid arthritis	5	0.0256
601	Glycosphingolipid biosynthesis - lacto and neolacto series	3	0.0391
4721	Synaptic vesicle cycle	4	0.0408
5120	Epithelial cell signaling in Helicobacter pylori infection	4	0.0495

**Fig. 3:**
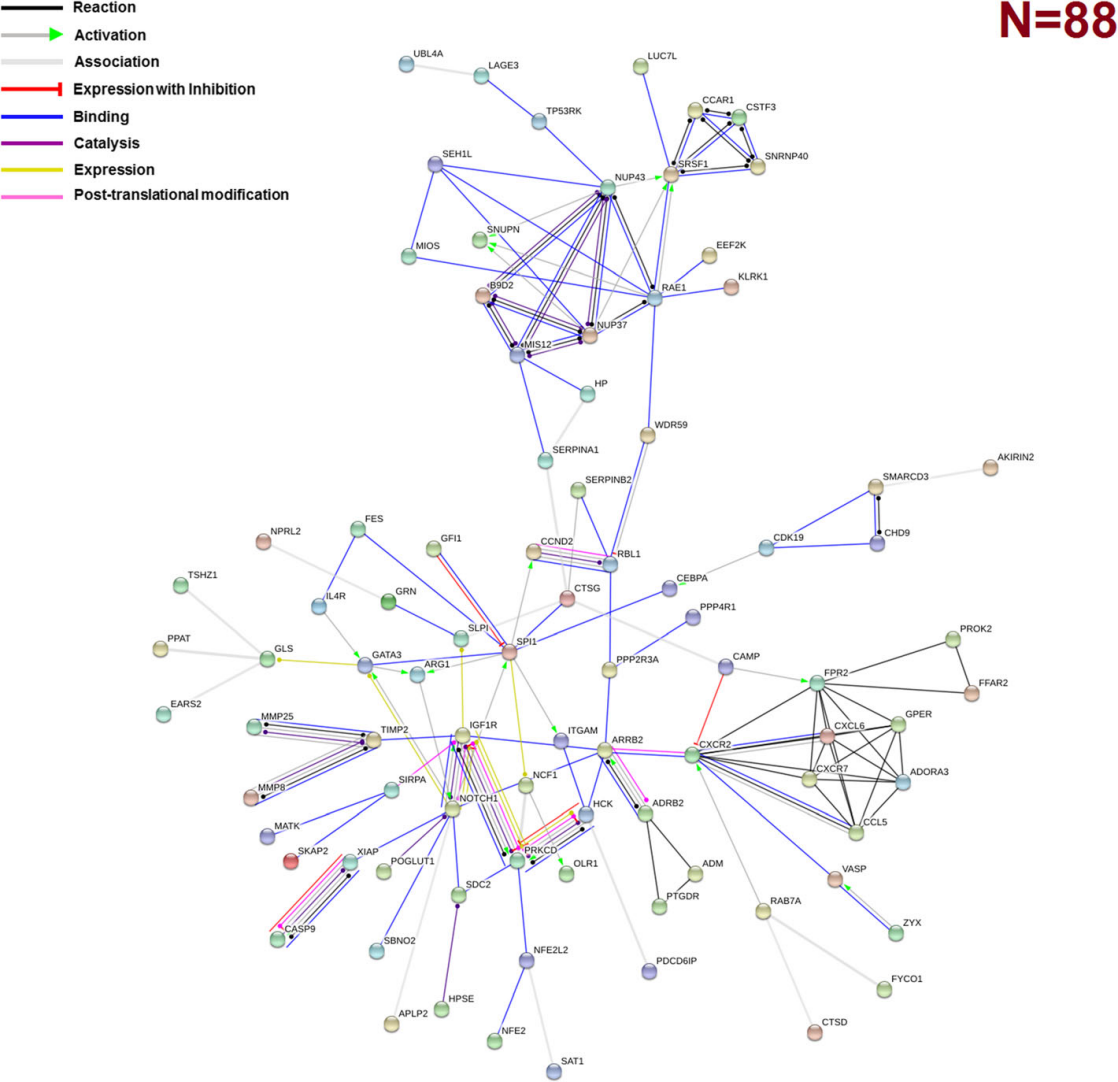
Gene/protein association network of the 88 MI DEGs displayed in the action view. Lines of different colors indicate predicted modes of action shown in the inset with a confidence interaction cut-off score of 0.7. The network was constructed using STRING

The possible interactions among the 626 DEGs were further investigated and visualized using STRING. We identified 88 gene products that were strongly interconnected and formed a network at a high confidence level (Fig. 3). Proteins are represented as nodes and the associations are denoted by edges (lines), corresponding to various molecular modes of action. KEGG pathway analysis of these genes identified genes of the Proteasome complex, and genes involved in RNA transport, endocytosis, phagocytosis, glycerophosholipid metabolism and glycosaminoglycan biosynthesis (Table 2C). Proteins with more than six interacting partners at a confidence interaction score of 0.7 were considered as ‘hubs’ of the network and were selected for further analysis (Additional file 1: Table S5). These 15 genes/proteins appear to form two distinct subnetworks (Fig. 3). The first sub-network includes genes involved in inflammation while the second contains proteins responsible for RNA processing and nuclear import/export. The first sub-network includes ADORA3, ARRB2, CCL5, CXCL6, CXCR2, CXCR7, FPR2 and GPER, while the second one contains NUP37, NUP43, RAE1 and SRSF1 and the related genes CCAR1, CSTF3, SNRP40 or SEH1L, SNJPN, MIOS and B9D2. A third, less interconnected, much smaller sub-network consists of NOTCH1, IGF1R, and SPI1. Remarkably, SERPIN, WDR59, RBL1, and CTSG proteins appear as interconnecting nodes of the first two sub-networks (Fig. 3). The pathway enrichment analysis showed that among the genes corresponding to the 15 most highly connected nodes (Additional file 1: Table S5) there are three significantly enriched KEGG Pathways (Additional file 1: Table S6). Two of these pathways are related to immunity and inflammation (*ARRB2*, *CCL5*, *CXCL6*, *CXCR2* and *CXCR7*) and one pathway in RNA transport (*NUP37*, *NUP43* and *RAE1*). The 15 genes were also used in logistic regression model stratified by study (individual patients’ data meta-analysis [43]), in order to assess their ability to predict the outcome (i.e., MI). Notably, even though these genes were not selected using existing variable selection techniques, but instead through functional enrichment analysis, they proved to be rather good predictors for MI, since the resulting model yields 84% sensitivity and 86% specificity.

A comparison between the MI/CAD-associated genes and the 626 DEGs identified in the present study was also performed. A total of 221 genes were found to be robustly involved in CAD/MI (Additional file 1: Table S7) by analyzing a large dataset resulted from a previous comprehensive study of 3854 disease-associated genes [37, 38]. The overlap between the set of 626 DEGs and the 221 genetic association genes was, however, minimal since only eight common genes were found: FES, GPD1L, IMPA2, OLR1, PGS1, PPP1R3B, ST3GAL4 and ABCB1. Interestingly, these genes do not appear to be functionally related, since their corresponding nodes in the interaction network are not connected (Fig. 3). Of particular note, three of these genes are among the top 60 DEGs with an FDR less than 10^− 8^ (FES, ST3GAL4 and PPP1R3B). We also performed an enrichment analysis of the 221 MI/CAD-associated genes, using the same settings in order to examine whether they overlap with the 626 DEGs identified in this work (Additional file 1: Table S8). The results showed that there is some overlap since 6 out of the 14 biological processes of the 626 DEGs are common with those from the functional analysis of genetic association genes. Given that these processes are multifaceted (i.e., cellular process, single-organism cellular process, cellular metabolic process, metabolic process, primary metabolic process, single-organism process), it was expected to include nearly 50% of the identified genes. Notably, among the biological processes found in MI/CAD-associated genes with high significance are processes related to cholesterol and lipid molecular processes and response to stress. Such biological processes were not identified among the DEGs, in which inflammatory processes are common. Finally, DEGs and MI/CAD-associated genes participate in distinctly different biochemical pathways according to KEGG.

## Discussion

In this systematic review and meta-analysis, we combined, for the first time to our knowledge, all the available literature and microarray data on MI and performed a meta-analysis in order to identify differentially expressed genes that can potentially be utilized as risk prediction factors. One of the main problems concerning microarray experiments is the lack of standardization. As a result, the data collected from different microarray platforms cannot be compared accurately or replicated. In a recent evaluation study, it was found that a large proportion of published studies could not be reproduced either completely or partially [44]. This was mainly attributed to data unavailability and incomplete data annotation or specification of data processing and analysis. The authors called for stricter publication rules that would enable public data availability and explicit description of data processing and analysis. The issue of comparing data generated by different platforms has long been under investigation [45] and filtering of probes has been shown to significantly improve intra-platform data comparability [46]. Of note, the problem of data availability emerged also in this meta-analysis, since the systematic review that we performed identified one additional published study that met all the inclusion criteria but its data were not available. The list of DEGs identified by this study was, as expected, smaller and had little overlap with the list of 626 DEGs identified by the meta-analysis.

In this work, by applying formal statistical methodologies for meta-analysis, we identified 626 statistically significant DEGs. It is worth mentioning that approximately half of the genes identified in this meta-analysis could not have been detected by any individual study using the same criteria. These findings reinforce the robustness and the value of the meta-analysis in the field of high-throughput data analysis. Additionally, based on bioinformatics analyses we attained the visualization of the interactions among these genes/gene products, the identification of their biochemical pathways, their cellular topology and their gene ontology function.

Several methods for combining different datasets in a meta-analysis have been proposed which can help researchers to overcome some of the problems mentioned above [47]. However, issues such as the lack of standardization present important obstacles in the application of such methods. Several studies in the literature compare the different microarray meta-analysis methods [24, 48, 49]. Notably, the lack of standardization is also apparent in the literature pertinent to studies in the meta-analysis of microarrays, since different methods and combinations of these methods have been used in the recent literature. A recent systematic search in PubMed, resulted in the empirical evaluation of the articles that reported microarray meta-analysis [50]. The results of this evaluation were very interesting, since a large proportion of the published studies was found to be conducted using the so-called “inappropriate” method of pooling datasets. This is a well-known issue in the meta-analysis literature, and this approach of pooling datasets in order to simply create a larger one is not recommended, as it can lead to various types of bias. Inappropriate is also the so-called method of “vote counting”, in which genes are considered DEGs only if they are found to have statistical significant differences in expression in the majority of the published studies. The Cochrane Handbook for Systematic Reviews of Interventions [51], states precisely: “*Vote counting … should be avoided whenever possible*…(and that it) … *might be considered as a last resort in situations when standard meta-analytical methods cannot be applied*”. We need to mention that the comparison of the DEGs identified by single studies was performed precisely to make this point clear: single studies are underpowered and in a combined analysis many genes, that did not appear significant in any study, may show differential expression.

Moreover, bioinformatics analysis revealed a rather small set of 88 highly interconnected genes/gene products identified as differentially expressed in MI. Based on metabolic pathway analysis, these genes are implicated into inflammatory/thrombotic/wound healing processes and RNA transport. The first sub-network consists of the genes ADORA3, ARRB2, CCL5, CXCL6, CXCR2 (IL8RB), CXCR7, FPR2 and GPER. Of those, ADORA3, CXCR2, CXCR7, FPR2 and GPER are G protein-coupled receptors (GPCRs), while the rest (ARRB2, CCL5, CXCL6) are ligands for GPCRs. Of particular note, MI mainly results from atherosclerosis, a disease manifested by chronic inflammatory response of white blood cells (WBCs) in the walls of arteries [52]. Platelets are shown to play a pivotal role in atherogenesis. Many platelet-derived chemokines can alter the differentiation of T-cells and macrophages by inhibiting neutrophil and monocyte apoptosis, or by triggering atherogenic monocyte recruitment on endothelium cells such as CXCL4 and CCL5. However, other chemokines display atheroprotective activity such as CXCL12, the ligand of CXCR7. CXCL12 has angiogenic properties [53, 54], since it is involved in regenerative processes by attracting progenitor cells and accelerating endothelial healing after injury [55]. ARRB2 is implicated in IL8-mediated granule release in neutrophils [56]. Ligand FPR2 (FPRL1) acts as a powerful chemotactic factor/agent for neutrophils. GPER is activated by the female sex hormone estradiol and plays a cardioprotective role by reducing cardiac hypertrophy and perivascular fibrosis. The aforementioned proteins, which belong to the first sub-network, are all ligands or receptors, mainly involved in chemokine signaling, and constitute a fine tuned network that regulates the atherogenetic or atheroprotective processes before, during and after MI [52, 57].

A smaller sub-network including NOTCH1, PRKCD, IGF1R, and SPI1 connected to the previous sub-network via ARRB2 is also formed (Fig. 3). NOTCH1 and IGF1R are transmembrane receptors. PRKCD is a Calcium-independent serine/threonine-protein kinase and regulates platelet functional responses. On the other hand, SPI1 is a transcriptional activator that may be specifically involved in the differentiation or activation of macrophages or B-cells; it also binds RNA and may modulate pre-mRNA splicing [33]. Another major subgroup consists of NUP37, NUP43, RAE1 and SRSF1 that are connected to CCAR1, CSTF3, SNRP40 or SEH1L, SNJPN, MIOS and B9D2. These genes/gene products are involved in RNA processing, transport and localization, cell cycle regulation as well as in glucose transport. Four of these proteins are implicated in the mitotic envelope disassembly and almost all of them are localized on nuclear membrane and especially on nuclear pores [33]. RNA transport and nuclear pore genes have not been proposed to be associated with MI. To our knowledge, it is the first time that such a mechanism/pathway is suggested to be involved in the development or recovery of MI.

Finally, we should mention five genes that constitute intermediate nodes between the two major sub-networks, the cytokine-receptor inflammatory genes and the transport genes. These are SERPINA1, SERPINB2, WDR59, RBL1 and CTSG. They are linearly connected to each other in a path (Fig. 3). Of those, two are serpin peptidase inhibitors (SERPINA1, SERPINB2), while CTSG is a serine protease with trypsin- and chymotrypsin-like specificity. WDR59 is a component of the GATOR sub-complex that functions as an activator of the amino acid-sensing branch of the TORC1 pathway. RBL1, retinoblastoma like 1 protein, is involved in the regulation of entry into cell division. Of note, the 4G/5G polymorphism of SERPINE1, another serpin peptidase inhibitor, has been shown in a meta-analysis conducted by Tsantes et al. to be significantly associated with MI and venus thrombosis [58]. The fact that our meta-analysis method identified genes known to be associated with MI highlights the importance of the novel finding of this study which is the involvement of RNA transport genes in MI.

Of particular importance, the genes found to be differentially expressed in MI in this study, or the subset of these genes that form the functional network, are not the same as the genes carrying polymorphisms which were previously identified in genetic association studies or GWAS [59]. Only eight out of 626 genes were common with those identified by genetic association studies. This is of no surprise, since GWAS mainly identify genes the polymorphisms of which are associated with the disease, whereas microarray studies, such as the ones included here, identify genes differentially expressed in the disease (and in particular, in blood). Genes of the former category are more likely to be the initiators of the disease (i.e. a transcription factor, a non-functional enzyme in metabolism and so on), whereas genes of the latter category are more likely to participate in subsequent events in the progression of the disease (indicators of the manifestation of the disease and so on). This is also exemplified in the enrichment analysis performed, which showed that DEGs participate mainly in inflammatory processes, whereas MI/CAD-associated genes participate mainly in lipid and cholesterol metabolism processes. The eight common genes are involved in lipid metabolism (GPD1L, OLR1, PGS1), membrane transport and signaling cascade (FES, IMPA2 and ABCB1), as well as glycogen and glucose metabolism (PPP1R3B, ST3GAL4). Of importance, three of these genes, namely, FES, ST3GAL4 and PPP1R3B, rank among the top 66 genes with the highest strength of association (smaller *q*-value). Despite the small number of common genes, this finding reflects the way gene polymorphisms and their corresponding proteins contribute to the development of cardiovascular lesions that eventually lead to MI. The eight genes common in both approaches, should be considered important since for these genes we know that they have variants associated with the disease and at the same time they are differentially expressed in the disease and should be investigated further.

This meta-analysis has certain limitations that should be acknowledged. First of all, public microarray data are often poorly annotated with respect to the outcome of patients after a primary myocardial infarction event. Second, we concentrated on blood samples taking into consideration the potential application of the identified DEGs as MI biomarkers. Gene expression data from other tissues, such as myocardium, muscle or liver, might have provided a different insight regarding the aetiology and the progression of the disease. However, such data are not readily available and are not likely to be used in clinical practice. Third, the use of microarray technology in studying gene expression is being surpassed by RNAseq, a method that provides a potentially more accurate quantification of the abundance of different transcripts; however, there are currently no available data on MI.

Nevertheless, the use of meta-analysis is required more than ever for the extraction of meaningful information contained in the huge amount of gene expression data that have been produced and stored in public repositories. In terms of methodology the present study has certain strengths. First, we retrieved all the publicly available microarray datasets on MI patients. Second, we applied several well-documented statistical techniques in the meta-analysis of these data and were able to identify sets of genes that are differentially expressed and could not be detected in the individual microarray studies. Third, bioinformatics approaches allowed us to gain important insight into the network formed by these particular genes/gene products. The increasing number of microarray datasets poses the need for the efficient management, processing, analysis, interpretation and clinical utility of these data. The combination of genetic risk factors with gene expression profiles and traditional risk predictors, such as Framingham score, may potentially provide a more accurate risk prediction model for identifying people at high risk for death after MI. They could also enable personalized treatment and health providers to make effective clinical decisions.

## Conclusions

In summary, in this comprehensive meta-analysis we identified a total of 626 genes that are differentially expressed between MI patients and healthy individuals. Based on functional enrichment analyses, DEGs were shown to be mainly involved in inflammatory/wound healing, RNA processing/transport mechanisms and a yet not fully characterized pathway involved in RNA transport and nuclear pore proteins. Moreover, there was a minimal overlap of these genes with genes identified by genetic association studies, but among these there are genes involved in lipid metabolism (GPD1L, OLR1, PGS1), membrane transport and signaling cascade (FES, IMPA2 and ABCB1), and glycogen and glucose metabolism (PPP1R3B, ST3GAL4). These data could be useful in future studies on the molecular mechanisms of MI as well as in the clinical setting as diagnostic and prognostic markers.

## Additional files

Additional file 1: This file includes the Meta-analysis Prisma flowchart and the supplementary results regarding the data analysis of the article. (DOCX 756 kb)

Additional file 2: PMIDs for each article and the excluding reasons. This file provides a detailed list of PMIDs for the articles identified and the reasons for their exclusion. (TXT 3 kb)

## Abbreviations

CAD: Coronary Artery Disease
DEGs: Differentially Expressed Genes
FDR: False Discovery Rate
FWER: Family-Wise Error Rate
GEO: Gene Expression Omnibus
IDR: Integration-driven Discovery Rate
IHD: Ischemic Heart Disease
MI: Myocardial Infarction
OMIM: Online Mendelian Inheritance in Man
SNPs: Single Nucleotide Polymorphisms
STRING: Search Tool for the Retrieval of INteracting Genes/proteins
WebGestalt: WEB-based GEne SeT AnaLysis Toolkit
