# Supplementary material for: Identification of gene expression profiles in myocardial infarction: a systematic review and meta-analysis
Source: BMC Med Genomics. 2018 Nov 27;11:109. doi: 10.1186/s12920-018-0427-x (PMC6260684; doi:10.1186/s12920-018-0427-x)
Supplement: Supplementary file 1 — This file includes the Meta-analysis Prisma flowchart and the supplementary results regarding the data analysis of the article. (DOCX 756 kb) [file 12920_2018_427_MOESM1_ESM.docx]

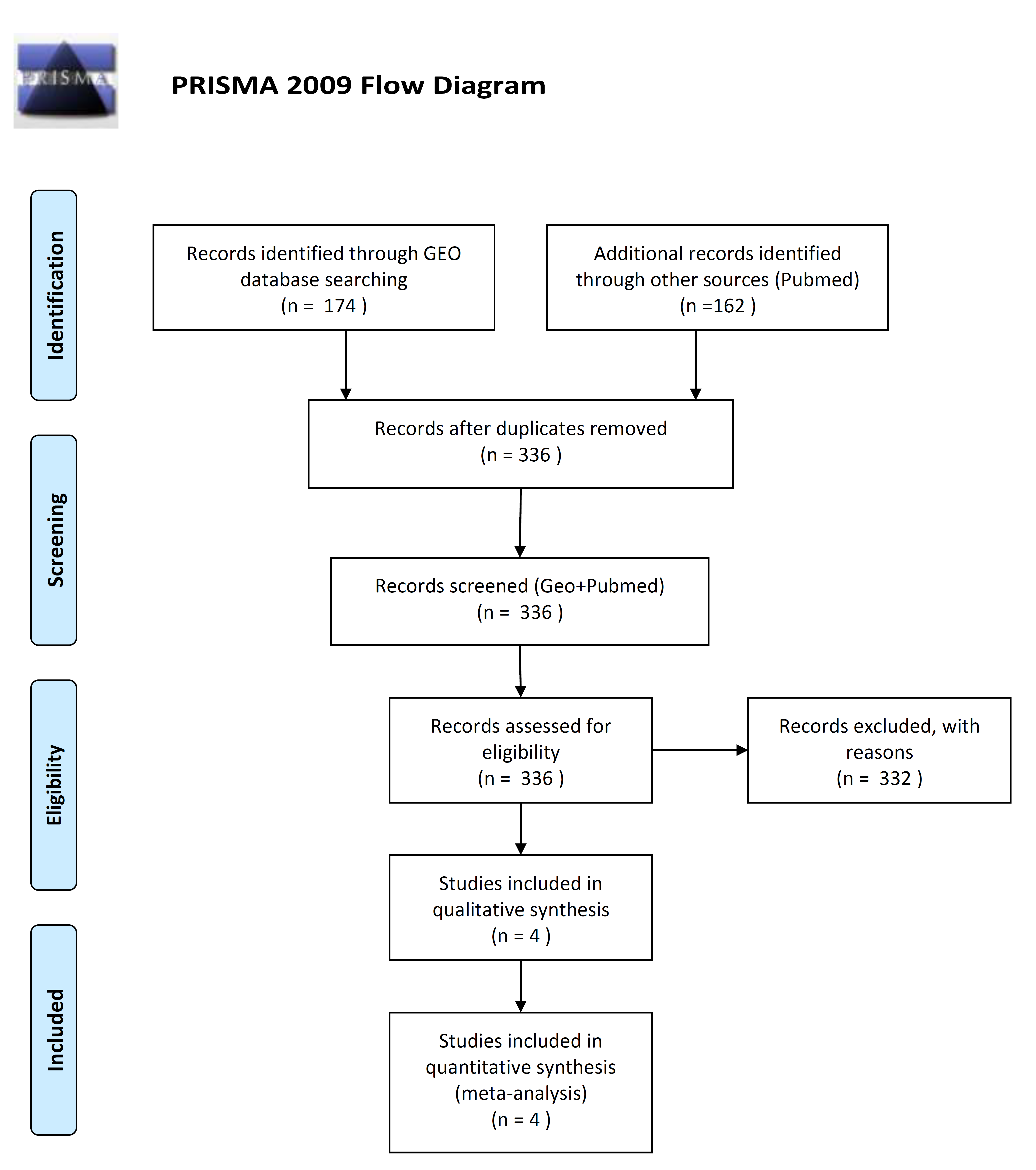


**Figure S1. Meta-analysis Prisma flowchart**

**Table S1. The 26 DEGs identified by Głogowska-Ligus J, Dąbek J (2012). Genes with * identified statistically significant also in our meta-analysis.**

| ATCB | HLA | C9orf16 |
| --- | --- | --- |
| PSAP | CD14 | IMPG1 |
| RPL18A | CD52 | RPL3 |
| H2A | APP | FTH |
| HSPA8 | TKT* | DEFA1 |
| HCK* | PKM | GAPDH |
| SERPINA1* | HBA1 | SMT3 |
| PPIB | VIM | S100A9 |
| TGFB1 | FCN1 |  |

**Table S2. Number of statistical significant genes per multiple correction method.**

|  | **p-value<0.05** | **FDR<0.01** | **Bonferroni** | **Holm** | **Sidak** | **Holland** |
| --- | --- | --- | --- | --- | --- | --- |
| **GSE48060** | 2845 | 3 | 3 | 3 | 3 | 3 |
| **GSE60993** | 4250 | 112 | 15 | 15 | 15 | 15 |
| **GSE61144** | 5916 | 2198 | 260 | 263 | 263 | 268 |
| **GSE66360** | 4694 | 1170 | 325 | 325 | 325 | 325 |
| **Meta-analysis** | 4306 | 626 | 158 | 158 | 160 | 160 |

**Table S3. The 626 Differentially Expressed Genes; FDR < 0.01**

| MMP25 | LOC642684 | EPHB4 | BXDC1 | PHYH2 | THEDC1 | LOC402571 |
| --- | --- | --- | --- | --- | --- | --- |
| S100A8 | KRT23 | C10ORF38 | SDCCAG3 | DPEP3 | IMMT | FLJ20512 |
| QIL1 | CBFA2T2 | SBK1 | TMEM103 | SLFN13 | MNDA | LILRA1 |
| ADRB2 | TUSC4 | C17ORF60 | FLJ10769 | TNFRSF12A | GATA3 | CAMSAP1 |
| CEACAM3 | LOC644128 | FLJ31413 | LTBR | LIMK2 | IL1RN | C7ORF25 |
| CAMP | MRPS10 | DET1 | GLS | MUTED | MUM1 | MRPS25 |
| SLC19A1 | LOC339123 | CITED4 | LOC221143 | FHL1 | RSHL2 | ACPP |
| LAX1 | LOC400924 | ZNF537 | MCEMP1 | ABCB1 | CLEC3B | ZBTB4 |
| DXS9879E | MEFV | PLA2G12A | ORF1_FL49 | LOC283547 | C6ORF130 | LOC644850 |
| LOC649986 | PXMP4 | NAG6 | ELOVL6 | MTP18 | DOK3 | LOC649095 |
| CHST12 | HRIHFB2122 | C14ORF112 | RAB7 | NFE2 | NLGN3 | IKZF2 |
| ATP6V0E2L | RPP21 | IL8RB | SLC12A6 | DCLRE1A | FLJ38984 | SRSF1 |
| PGLYRP1 | SLPI | LOC644039 | VASP | FLJ20551 | TREML3 | TTC13 |
| CKAP1 | GLT1D1 | PPAT | KIAA1026 | TSPAN32 | LOC650795 | CCNJL |
| FES | GALNAC4S_6ST | ARRB2 | GPR27 | FCGR1A | MLF2 | SLC22A14 |
| HK3 | ZNF234 | TFB1M | SAT | MGA | WDR71 | KSP37 |
| PDXP | FCRL6 | FLJ22662 | TIMM17B | PITPNA | GLA | LTF |
| RBL1 | DFFA | HYLS1 | SIP1 | BBS2 | TXK | ALKBH8 |
| EVA1 | RPA1 | MGC18216 | FLJ33641 | PDZD3 | SLC5A9 | HSPA6 |
| HMG20A | CROT | IMPA2 | LOC441034 | FAM26B | MSL3L1 | TASP1 |
| HCK | GTPBP6 | C1QR1 | ZNF573 | CAMTA1 | SIGLEC5 | LOC51035 |
| SLC16A3 | C3ORF31 | ERF | FAM27E3 | RNASE4 | CLEC1A | PHACTR4 |
| OACT1 | TP53INP1 | POGK | C17ORF42 | TRIM73 | ARV1 | GPR177 |
| LOC651738 | SLC6A16 | GCM1 | ATP6V0B | ZCSL2 | WDR89 | PDDC1 |
| NCALD | LBH | KIAA0999 | TSEN54 | GPD1L | MMP8 | RTP4 |
| PLEKHG4 | CXCR7 | HIBADH | DYSF | CKAP4 | SEPT11 | NUP37 |
| PAFAH2 | RAE1 | ZNF364 | SPIN3 | SERTAD3 | ZFP90 |  |
| LOC653610 | HSPC176 | DBT | LIN7A | DYRK2 | EXOSC6 |  |
| EIF3S7 | RAB43 | GBA | ORM1 | ZBTB40 | SAP30 |  |
| MGC7036 | EIF3S6IP | C1ORF108 | DENND1A | SEH1L | DPY19L4 |  |
| ZYX | KDELC2 | FLOT1 | RAB33A | KLRG1 | FLJ45445 |  |
| UBL4A | XAB1 | ZNF302 | PNMA1 | TCEAL8 | PIGL |  |
| LOC652878 | C4ORF29 | IL4R | PROK2 | FFAR2 | B3GALT7 |  |
| HMFN0839 | NIFIE14 | MGC4562 | SCAP2 | TBC1D2 | SDC2 |  |
| CTSD | RNUT1 | METT10D | TNFAIP8L1 | PGD | PYHIN1 |  |
| LOC650761 | SYNJ2BP | C1ORF183 | TLR5 | TMEM41A | TOX |  |
| MRPS5 | VPS45A | CSF2RB | LRRC39 | ADORA3 | CCL5 |  |
| TRA16 | ST3GAL6 | COP1 | LOC440731 | MIMITIN | LOC202781 | |
| ADM | FPRL1 | ZNF467 | LOC648716 | IBRDC3 | TNFSF5IP1 | |
| ANXA3 | MMS19L | TM4SF19 | RBM28 | RHOG | KLHL6 |  |
| UST | DFFB | MGC4093 | PCSK7 | OSBPL3 | HPSE |  |
| C18ORF17 | COQ10A | DJ341D101 | HOMER3 | FAM20B | KIAA1434 | |
| FLJ10081 | LOC642161 | PSMB2 | CEBPA | NRBP1 | YPEL1 |  |
| ST3GAL4 | NUAK2 | MIS12 | ZNF519 | RAB11FIP3 | ABCA13 |  |
| TRIB2 | NAPEPLD | HEG1 | LOC641825 | DNAJB12 | ZFPL1 |  |
| CCDC76 | COLQ | SERPINB8 | PDE6B | ZNF500 | LRRC25 |  |
| TAF15 | LUC7L | ZNF550 | CUL4A | C9ORF71 | LOC100287808 | |
| ALPL | HIST1H3D | PRF1 | THAP11 | LOC654053 | IDI1 |  |
| TCN1 | DHFRL1 | PBEF1 | PTPNS1L3 | LOC647000 | FLJ12788 |  |
| WDR57 | LOC642755 | PSCDBP | LOC100499466 | PANX2 | ICK |  |
| CRYZL1 | STEAP4 | LOC100190986 | NIF3L1 | MARK2 | C10ORF54 | |
| KIAA0701 | LOC644869 | LOC284648 | LOC652595 | C6ORF26MSH5 | SH2D3A |  |
| PPP1R3B | PPP4R1 | IL7R | SPI1 | LOC152485 | FAM113A |  |
| PTPNS1 | WDR59 | CRR9 | MGC40499 | PLEKHA1 | RNF141 |  |
| KLRK1 | LOC349114 | SCRN1 | SERPINB2 | LOC399900 | NEK1 |  |
| TRAF3IP3 | SULT1B1 | FLJ20643 | C9ORF5 | ZNF438 | CCND2 |  |
| LAT | NFE2L2 | LOC100506828 | CSTF3 | TRIM61 | MGC13096 | |
| IBRDC2 | IKIP | LOC650472 | MCEE | FAM100B | LY96 |  |
| LOC440926 | GPX3 | TARP | FLJ21749 | NCRNA00182 | ATP5S |  |
| SNAPC3 | MGC3121 | TSPAN31 | PHF15 | LOC643284 | IL17R |  |
| B3GNT5 | CA4 | BPI | SIGLEC9 | AQP9 | SDCCAG33 | |
| CRYGS | LOC647099 | GPR89A | VEZT | STXBP2 | RAD17 |  |
| MATK | FLJ20272 | KLRB1 | ARMET | LOC652615 | FLJ37307 |  |
| ZCCHC17 | CD177 | LOC284393 | FLJ20699 | LOC285053 | CLTCL1 |  |
| PRKCD | HP | ATF7IP2 | FLJ10241 | TP53RK | FLJ14166 |  |
| NOLA2 | AIFM3 | SAMD3 | CDC2L6 | NICN1 | LOC650737 | |
| NP | FLJ11259 | TMTC4 | PRSS15 | ZNF567 | GPR30 |  |
| PTGDR | LOC645625 | OPLAH | PDCD6IP | LOC401019 | PGS1 |  |
| TMEM88 | GRN | ABI2 | S100A12 | CXCL6 | ITGAM |  |
| ZNF785 | LOC652626 | NMT2 | RRAS2 | KLHDC5 | DDEF1 |  |
| SYTL2 | KIAA1970 | TMCO3 | LOC652025 | MTX1 | ZNRD1 |  |
| CECR6 | PHC2 | CPEB2 | DKFZP564J157 | C11ORF46 | VNN2 |  |
| WDR67 | EIF3S2 | C1ORF33 | GFI1 | LIMA1 | PIGN |  |
| LOC653314 | LOC440093 | ZNF545 | ZDHHC18 | M_RIP | LOC399744 | |
| SLAMF7 | CARD12 | LOC136143 | C9ORF74 | RAB2 | PARP2 |  |
| CHD9 | NCF1 | DAZAP2 | DDX56 | C16ORF24 | MAT2A |  |
| C3ORF9 | MGC13170 | MGC27345 | EEF2K | KIF13A | TAF1B |  |
| PDCD5 | HSPC196 | ECE1 | DNAJC24 | LOC387841 | ZNF207 |  |
| KIAA0963 | GZMA | PCNT2 | SIRPD | LOC440348 | XIAP |  |
| SEC22L1 | TGM3 | APOL3 | LOC391766 | PLRG1 | TRPM6 |  |
| C6ORF166 | LSM11 | LCMT2 | BASP1 | PLEKHA9 | ZMYM6 |  |
| MOSC1 | APLP2 | AGAP1 | NPAT | LOC347292 | FLJ20323 |  |
| GALNACT_2 | SCAP1 | IMPDH1 | C9ORF19 | PHC1 | LOC642233 | |
| FLJ22471 | NINJ1 | LOC285550 | TRIM68 | KIAA0446 | BCOR |  |
| APOBEC3G | C22ORF16 | CLEC5A | LOC57228 | CLIC3 | STARD10 |  |
| ARMC1 | STATIP1 | PYCR2 | MLL | LOC654052 | PCCA |  |
| FLJ10379 | RASSF3 | MPHOSPH1 | RABEP1 | CYP27A1 | LOC651143 | |
| DGAT2 | PTPN4 | TMEM117 | HSPC117 | FYCO1 | DAG1 |  |
| EIF2B2 | NUP43 | CASP9 | KIAA0182 | LOC144571 | CD28 |  |
| SMARCAD1 | C9ORF111 | COL18A1 | ZMYND11 | TIMM22 | CTSG |  |
| TKT | NOTCH1 | FLJ21945 | TIMP2 | GPM6B | MTX2 |  |
| GIMAP6 | ARL13B | TXNDC14 | SERPINA1 | NGFRAP1 | CYP20A1 |  |
| NPAL3 | THOC3 | BTBD14A | C15ORF39 | MRPS11 | TMEM119 |  |
| DKFZP434K1815 | MYO9A | AGTRAP | HADH2 | MGC15763 | PLSCR1 |  |
| C19ORF12 | NR2C1 | HOXB2 | PLB1 | USP37 | CORO1C |  |
| TRAPPC2 | OLR1 | LOC653337 | SLC30A7 | ZNF562 | AKAP11 |  |
| FEZ1 | ST3GAL5 | SMARCD3 | C21ORF127 | LY6G6C | PPCDC |  |
| ARG1 | TNFSF13 | STX3A | PPP2R3A | CLEC2D | ANKRD13 | |
| MGC2463 | KREMEN1 | SLC7A6 | IKZF3 | GCA | CCAR1 |  |
| TNRC5 | TXNDC13 | ECHDC3 | JTV1 | HRBL | SMPDL3A | |

**Table S4. Top 60 Differentially Expressed Genes; FDR < 10^-8^**

| MMP25 | HCK | UST |
| --- | --- | --- |
| S100A8 | SLC16A3 | C18ORF17 |
| QIL1 | OACT1 | FLJ10081 |
| ADRB2 | LOC651738 | ST3GAL4 |
| CEACAM3 | NCALD | TRIB2 |
| CAMP | PLEKHG4 | CCDC76 |
| SLC19A1 | PAFAH2 | TAF15 |
| LAX1 | LOC653610 | ALPL |
| DXS9879E | EIF3S7 | TCN1 |
| LOC649986 | MGC7036 | WDR57 |
| CHST12 | ZYX | CRYZL1 |
| ATP6V0E2L | UBL4A | KIAA0701 |
| PGLYRP1 | LOC652878 | PPP1R3B |
| CKAP1 | HMFN0839 | PTPNS1 |
| FES | CTSD | KLRK1 |
| HK3 | LOC650761 | TRAF3IP3 |
| PDXP | MRPS5 | LAT |
| RBL1 | TRA16 | IBRDC2 |
| EVA1 | ADM | SNAPC3 |
| HMG20A | ANXA3 | LOC440926 |

**Table S5. The 15 most highly connected nodes at a confidence cut-off score of 0.7.**

| **Symbol** | **Name** | **Number of links** |
| --- | --- | --- |
| **CXCR2** | C-X-C motif chemokine receptor 2 | 10 |
| **NOTCH1** | notch 1 | 10 |
| **SPI1** | Spi-1 proto-oncogene | 10 |
| **FPR2** | formyl peptide receptor 2 | 9 |
| **RAE1** | ribonucleic acid export 1 | 9 |
| **NUP43** | nucleoporin 43 | 8 |
| **NUP37** | nucleoporin 37 | 7 |
| **SRSF1** | serine and arginine rich splicing factor 1 | 7 |
| **CXCR7** | chemokine (C-X-C motif) receptor 7 | 6 |
| **CCL5** | C-C motif chemokine ligand 5 | 6 |
| **CXCL6** | C-X-C motif chemokine ligand 6 | 6 |
| **ADORA3** | adenosine A3 receptor | 6 |
| **GPER** | G protein-coupled estrogen receptor 1 | 6 |
| **ARRB2** | arrestin beta 2 | 6 |
| **IGF1R** | insulin like growth factor 1 receptor | 6 |

**Table S6. Over-represented KEGG Pathway terms of the 15 most highly connected nodes listed in eTable 4; adjusted p-value ≤ 0.001**

| **KEGG Pathway Name** | **Gene Symbol** | **adjusted P-value** |
| --- | --- | --- |
| **Cytokine-cytokine receptor interaction** | CCL5 CXCR7 CXCR2 CXCL6 | 7.80e-05 |
| **Chemokine signaling pathway** | CCL5 ARRB2 CXCR2 CXCL6 | 0.0003 |
| **RNA transport** | NUP43 NUP37 RAE1 | 0.0003 |

**Table S7. The 221 MI/CAD-associated genes. Genes with * are found statistical significant differentially expressed in our meta-analysis.**

| ABCA1 | CYP1A1 | LGALS2 | PPP3CA |
| --- | --- | --- | --- |
| ABCA8 | CYP1A2 | LIPC | PSRC1 |
| ABCG5 | DNAH11 | LPA | PTPN11 |
| ABCG8 | DNM2 | LPAL2 | PVRL2 |
| ABO | DOCK6 | LPL | RAB3GAP1 |
| ACAD10 | DOCK7 | LRP4 | RAF1 |
| ACE | DTNB | LRP6 | RETN |
| ADAMTS7 | EBF1 | LRP8 | RNF130 |
| ADH7 | EDC4 | LTA | RTKN2 |
| ADRB1 | ESR1 | MACROD2 | RYR2 |
| ALDH2 | ETFA | MAP4 | SBNO1 |
| ANGPTL4 | EVI5 | MAP4K5 | SCARB1 |
| ANKS1A | FADS1 | MECOM | SERPINA10 |
| APOA5 | FADS2 | MEF2A | SERPINE1 |
| APOB | FADS3 | MIA3 | SH2B3 |
| APOC1 | FES | MIAT | SLC12A9 |
| APOE | FIGN | MLXIPL | SLC22A3 |
| ARHGAP42 | FLT1 | MMAB | SLC39A8 |
| ARID5B | FNDC1 | MOV10 | SLC4A7 |
| ARL15 | FRK | MRAS | SMAD3 |
| ATP2B1 | FSTL4 | MSRA | SMARCA4 |
| ATXN2 | FUT2 | MTAP | SMG6 |
| BANK1 | GALNT2 | MTHFD1L | SP6 |
| BTNL2 | GCLC | MTHFR | SPTY2D1 |
| BUD13 | GCLM | MYL2 | SRR |
| C10ORF107 | GFOD2 | MYLIP | ST3GAL4 |
| C12ORF43 | GMPR | MYO16 | STAB2 |
| C4ORF22 | GNAS | MYO1H | STARD3 |
| C6ORF10 | GOSR2 | NCAN | STK32B |
| C6ORF106 | GPAM | NIPSNAP3B | STK39 |
| C9ORF3 | GUCY1A3 | NLRC5 | TBX3 |
| CACNB2 | HABP2 | NOS3 | TBX5 |
| CAMK4 | HAVCR1 | NPC1L1 | TCF21 |
| CAPZA1 | HCG27 | NPR3 | TCF7L2 |
| CASZ1 | HECTD4 | NPRL3 | TIMD4 |
| CCL2 | HERPUD1 | NT5C2 | TMEM57 |
| CD36 | HFE | NT5DC3 | TNFSF4 |
| CDH13 | HHIPL1 | NUMB | TNIK |
| CDKN2A | HLA-C | OAS3 | TOMM40 |
| CDKN2B | HMGCR | OLR1 | TOP1 |
| CELSR2 | HNF1A | OPRM1 | TRPS1 |
| CETP | HNF4A | PABPC4 | TTC39B |
| CHRM3 | HPR | PBX4 | UBE2L3 |
| CMIP | IGSF5 | PCSK9 | UBE2Z |
| CNNM2 | IRS1 | PDE3A | ULK4 |
| CNTN4 | ITGB3 | PDGFD | UMOD |
| COBLL1 | JAG1 | PGS1 | UTP20 |
| COL4A1 | KCNMB1 | PHACTR1 | WDR12 |
| COL4A2 | KCTD10 | PLEKHA7 | ZBED1P1 |
| CR1L | KIAA1462 | PLEKHO2 | ZC3HC1 |
| CSK | KL | PLTP | ZFAT |
| CSMD1 | KLHL29 | PON1 | ZFHX3 |
| CUX2 | LACTB | PON2 | ZNF652 |
| CX3CR1 | LAMC2 | PPAP2B | ZNF664 |
| CYP17A1 | LDLR | PPP1R3B | ZNF831 |
|  |  |  | ZPR1 |

**Table S8. Enrichment Analysis of the 221 MI/CAD-associated genes according to STRING.**

**Table S8A:** Functional enrichment of the 221 MI/CAD-associated genes for Biological Processes according to STRING. The color indicates the pathways that are common with the pathways of the 626 DEGs.

| #pathway ID | pathway description | observed gene count | false discovery rate |
| --- | --- | --- | --- |
| GO.0030301 | cholesterol transport | 15 | 3.90E-15 |
| GO.0055088 | lipid homeostasis | 17 | 8.20E-13 |
| GO.0097006 | regulation of plasma lipoprotein particle levels | 13 | 2.80E-12 |
| GO.0051239 | regulation of multicellular organismal process | 64 | 1.40E-11 |
| GO.0042632 | cholesterol homeostasis | 13 | 9.10E-11 |
| GO.0006869 | lipid transport | 19 | 3.10E-09 |
| GO.0044707 | single-multicellular organism process | 97 | 5.60E-09 |
| GO.0008203 | cholesterol metabolic process | 14 | 5.60E-09 |
| GO.0010876 | lipid localization | 19 | 1.40E-08 |
| GO.0034381 | plasma lipoprotein particle clearance | 8 | 1.70E-08 |
| GO.0009893 | positive regulation of metabolic process | 72 | 2.60E-08 |
| GO.2000026 | regulation of multicellular organismal development | 44 | 3.60E-08 |
| GO.0048731 | system development | 72 | 5.90E-08 |
| GO.0031325 | positive regulation of cellular metabolic process | 62 | 8.70E-08 |
| GO.0006898 | receptor-mediated endocytosis | 16 | 8.70E-08 |
| GO.0006950 | response to stress | 68 | 3.70E-07 |
| GO.0050793 | regulation of developmental process | 50 | 3.80E-07 |
| GO.0048522 | positive regulation of cellular process | 79 | 4.10E-07 |
| GO.0072358 | cardiovascular system development | 29 | 4.10E-07 |
| GO.0072359 | circulatory system development | 29 | 4.10E-07 |
| GO.0042592 | homeostatic process | 38 | 4.20E-07 |
| GO.0048518 | positive regulation of biological process | 86 | 6.50E-07 |
| GO.0044281 | small molecule metabolic process | 50 | 8.30E-07 |
| GO.0032368 | regulation of lipid transport | 11 | 8.90E-07 |
| GO.0034375 | high-density lipoprotein particle remodeling | 6 | 1.10E-06 |
| GO.0065009 | regulation of molecular function | 57 | 1.20E-06 |
| GO.0042060 | wound healing | 26 | 1.40E-06 |
| GO.0007399 | nervous system development | 47 | 1.60E-06 |
| GO.0010604 | positive regulation of macromolecule metabolic process | 56 | 1.70E-06 |
| GO.0032502 | developmental process | 81 | 1.70E-06 |
| GO.0051241 | negative regulation of multicellular organismal process | 31 | 1.70E-06 |
| GO.0009719 | response to endogenous stimulus | 38 | 2.00E-06 |
| GO.0031667 | response to nutrient levels | 20 | 2.40E-06 |
| GO.0048856 | anatomical structure development | 74 | 2.50E-06 |
| GO.0044767 | single-organism developmental process | 80 | 2.60E-06 |
| GO.0010033 | response to organic substance | 53 | 2.80E-06 |
| GO.0044093 | positive regulation of molecular function | 42 | 2.80E-06 |
| GO.0006639 | acylglycerol metabolic process | 11 | 3.20E-06 |
| GO.0044283 | small molecule biosynthetic process | 19 | 3.20E-06 |
| GO.0001944 | vasculature development | 21 | 3.30E-06 |
| GO.0043691 | reverse cholesterol transport | 6 | 3.30E-06 |
| GO.0019220 | regulation of phosphate metabolic process | 39 | 3.40E-06 |
| GO.0006066 | alcohol metabolic process | 18 | 3.60E-06 |
| GO.0071495 | cellular response to endogenous stimulus | 31 | 3.90E-06 |
| GO.0070328 | triglyceride homeostasis | 7 | 4.50E-06 |
| GO.0034372 | very-low-density lipoprotein particle remodeling | 5 | 5.80E-06 |
| GO.0034383 | low-density lipoprotein particle clearance | 5 | 5.80E-06 |
| GO.0001568 | blood vessel development | 20 | 6.20E-06 |
| GO.0010646 | regulation of cell communication | 57 | 6.20E-06 |
| GO.0044763 | single-organism cellular process | 133 | 7.70E-06 |
| GO.0006629 | lipid metabolic process | 31 | 9.50E-06 |
| GO.0071310 | cellular response to organic substance | 43 | 9.50E-06 |
| GO.0022008 | neurogenesis | 36 | 0.000014 |
| GO.0023051 | regulation of signaling | 54 | 0.000016 |
| GO.0006641 | triglyceride metabolic process | 10 | 0.000017 |
| GO.0044710 | single-organism metabolic process | 72 | 0.000018 |
| GO.0044699 | single-organism process | 133 | 0.00013 |
| GO.0023057 | negative regulation of signaling | 30 | 0.000133 |
| GO.0010899 | regulation of phosphatidylcholine catabolic process | 3 | 0.000136 |
| GO.0042493 | response to drug | 17 | 0.00014 |
| GO.0030154 | cell differentiation | 56 | 0.000152 |
| GO.0009653 | anatomical structure morphogenesis | 43 | 0.00016 |
| GO.0040011 | locomotion | 30 | 0.000185 |
| GO.0006720 | isoprenoid metabolic process | 9 | 0.000185 |
| GO.0048514 | blood vessel morphogenesis | 16 | 0.000185 |
| GO.0010874 | regulation of cholesterol efflux | 5 | 0.00019 |
| GO.0050896 | response to stimulus | 97 | 0.000234 |
| GO.0048583 | regulation of response to stimulus | 58 | 0.000254 |
| GO.0055091 | phospholipid homeostasis | 4 | 0.000261 |
| GO.0010883 | regulation of lipid storage | 6 | 0.000291 |
| GO.0006897 | endocytosis | 18 | 0.000296 |
| GO.0045937 | positive regulation of phosphate metabolic process | 26 | 0.000296 |
| GO.0009966 | regulation of signal transduction | 46 | 0.000354 |
| GO.0006721 | terpenoid metabolic process | 8 | 0.000373 |
| GO.0044241 | lipid digestion | 4 | 0.000383 |
| GO.0030100 | regulation of endocytosis | 11 | 0.000383 |
| GO.0034374 | low-density lipoprotein particle remodeling | 4 | 0.000383 |
| GO.0007154 | cell communication | 77 | 0.000387 |
| GO.0010648 | negative regulation of cell communication | 29 | 0.000393 |
| GO.0044711 | single-organism biosynthetic process | 31 | 0.000428 |
| GO.0045935 | positive regulation of nucleobase-containing compound metabolic process | 36 | 0.000437 |
| GO.0010817 | regulation of hormone levels | 12 | 0.000444 |
| GO.0010989 | negative regulation of low-density lipoprotein particle clearance | 3 | 0.000444 |
| GO.0009968 | negative regulation of signal transduction | 27 | 0.000444 |
| GO.2001237 | negative regulation of extrinsic apoptotic signaling pathway | 8 | 0.000444 |
| GO.0032496 | response to lipopolysaccharide | 13 | 0.000445 |
| GO.0019222 | regulation of metabolic process | 90 | 0.000447 |
| GO.0008152 | metabolic process | 117 | 0.00367 |
| GO.0065007 | biological regulation | 118 | 0.00462 |
| GO.0044237 | cellular metabolic process | 104 | 0.00852 |
| GO.0009987 | cellular process | 140 | 0.00922 |
| GO.0044238 | primary metabolic process | 103 | 0.0212 |

**Table S8B**: Cellular Component enrichment of the 221 MI/CAD-associated genes for Cellular Component according to STRING. The color indicates the pathways that are common with the pathways of the 626 DEGs.

| #pathway ID | pathway description | observed gene count | false discovery rate |
| --- | --- | --- | --- |
| GO.0005622 | intracellular | 146 | 0.0498 |
| GO.0005737 | cytoplasm | 120 | 0.0399 |
| GO.0044444 | cytoplasmic part | 93 | 0.0442 |
| GO.0005576 | extracellular region | 79 | 5.07E-06 |
| GO.0032991 | macromolecular complex | 70 | 0.00326 |
| GO.0044421 | extracellular region part | 69 | 1.42E-05 |
| GO.0071944 | cell periphery | 69 | 0.00623 |
| GO.0005886 | plasma membrane | 67 | 0.0108 |
| GO.0031982 | vesicle | 61 | 0.00122 |
| GO.0031988 | membrane-bounded vesicle | 59 | 0.00171 |
| GO.0070062 | extracellular exosome | 49 | 0.00449 |
| GO.0044459 | plasma membrane part | 40 | 0.0137 |
| GO.0005615 | extracellular space | 30 | 0.00136 |
| GO.0097458 | neuron part | 25 | 0.00623 |
| GO.0098589 | membrane region | 22 | 0.0393 |
| GO.0043005 | neuron projection | 19 | 0.0402 |
| GO.0009986 | cell surface | 18 | 0.0138 |
| GO.0036477 | somatodendritic compartment | 15 | 0.0498 |
| GO.0098552 | side of membrane | 14 | 0.00486 |
| GO.0043235 | receptor complex | 13 | 0.00122 |
| GO.0045177 | apical part of cell | 13 | 0.00449 |
| GO.0043025 | neuronal cell body | 13 | 0.0138 |
| GO.0034358 | plasma lipoprotein particle | 11 | 8.15E-11 |
| GO.0045121 | membrane raft | 11 | 0.00449 |

**Table S8C: KEGG Pathway terms of the 221 MI/CAD-associated genes.**

| #pathway ID | pathway description | observed gene count | false discovery rate |
| --- | --- | --- | --- |
| 4975 | Fat digestion and absorption | 8 | 1.88E-06 |
| 3320 | PPAR signaling pathway | 7 | 0.000735 |
| 4022 | cGMP-PKG signaling pathway | 10 | 0.000735 |
| 4976 | Bile secretion | 6 | 0.00604 |
| 4961 | Endocrine and other factor-regulated calcium reabsorption | 5 | 0.00708 |
| 4913 | Ovarian steroidogenesis | 5 | 0.00881 |
| 4921 | Oxytocin signaling pathway | 8 | 0.00916 |
| 4020 | Calcium signaling pathway | 8 | 0.0199 |
| 2010 | ABC transporters | 4 | 0.0266 |
| 4540 | Gap junction | 5 | 0.0497 |
| 4970 | Salivary secretion | 5 | 0.0497 |
